# Perceptions of and preferences for federally-funded family planning clinics

**DOI:** 10.1186/1742-4755-11-50

**Published:** 2014-06-30

**Authors:** Willie H Oglesby

**Affiliations:** 1 Department of Health Policy & Management, College of Public Health, Kent State University, PO Box 5190, 800 Hilltop Drive, 212 Moulton Hall, Kent, Ohio 44242, USA

**Keywords:** Family planning, Access to care, Sexual and reproductive health, Lifestyle medicine, Title X, HIV, STD, Affordable Care Act

## Abstract

**Background:**

The Title X family planning program provides affordable access to a range of sexual and reproductive health services, with a priority for low-income people. The disproportionate burden of unintended pregnancy, breast and cervical cancer, and sexually transmitted diseases among minority groups, teens, and young adults in the US underscore the need for affordable access to such services. However, increased access to sexual and reproductive health services, resulting from the Affordable Care Act (ACA) create questions regarding the continued need for this program.

**Methods:**

A study was conducted to assess clients’ perceptions of Title X-funded family planning clinics and their preferences for these clinics for a range of sexual and reproductive health services. An anonymous, self-administered, paper-and-pencil survey was administered to 696 clients who received services from one of eight Title X-funded family planning clinics in Northeast Ohio.

**Results:**

The majority of participants stated very positive perceptions of the Title X-funded clinics; that they “Always” go to the Title X-funded clinic for birth control, STD/HIV testing, and pregnancy testing; and that the Title X-funded clinic was their regular source of health care. Females were more likely than males to prefer the Title X clinic for birth control, physical exams, pregnancy testing, and health information and more teens under the age of 18 preferred to use the Title X clinic for STD/HIV testing, physical exams, pregnancy testing, and health information.

**Conclusions:**

Findings indicate that these Title X-funded family planning clinics successfully reached populations in need of sexual and reproductive health services and suggest that these facilities can help play an important role in reducing disparities even after full implementation of the Affordable Care Act. However, more research is needed to fully quantify the need and value of Title X-funded family planning clinics and its relation to the changing health care environment in the US.

## Background

Increased access to family planning services is regarded as one of the ten greatest public health achievements in the 20^th^ Century [1]. Family planning is comprised of a wide array of sexual and reproductive health services including contraceptive education and counseling; pregnancy testing and counseling; breast and cervical cancer screening; human immunodeficiency virus (HIV) testing; screening and treatment for sexually transmitted diseases (STDs); and other patient education and referrals [2].

### Need for sexual and reproductive health services

In 2006, nearly half (46%) of all pregnancies were unintended and these rates were highest among women 20–24 years of age, women with the fewest years of educational attainment, poor or low-income women, and Black women [3]. To help avoid unintended pregnancy, a wide range of contraception options are available including intrauterine contraception (e.g., IUDs), hormonal methods (e.g., implantable, injectable, and oral contraceptives), barrier methods (e.g., diaphragm and condom), and natural methods (e.g., basal body temperature, calendar, and cervical mucus methods), with varying levels of effectiveness [4].

Excluding skin cancers, breast cancer is the most common cancer among women [5-7] affecting approximately 230,000 women in 2013 [6]. From 2006 to 2010, the average annual female breast cancer incidence rate was highest for non-Hispanic white women (127.3 cases per 100,000 females) and lowest for Asian Americans/Pacific Islanders (84.7 cases per 100,000 females) [6]. Although non-Hispanic white women have the highest overall beast cancer incidence rates in most age groups, African American women have higher incidence rates among women younger than 40 years of age and have the highest breast cancer death rates (30.8 deaths per 100,000 females) [6]. The higher breast cancer death rate for African Americans, despite having lower incidence rates, is due to both a later stage disease at diagnosis and poorer state-specific survival [6]. In addition, researchers have pointed to differences in socioeconomic status as another driver of cancer death rates among different racial/ethnic groups [8].

Cervical cancer used to be the leading cause of cancer death among women in the United States, but due to increased screening using the Papanicolaou (Pap) test, the number of cervical cancer cases has steadily declined over the past 40 years [9]. Although regular Pap testing has contributed to decreasing cervical cancer incidence and mortality over the past decades, half of women diagnosed with cervical cancer have never had a Pap test [10-12]. Factors associated with not receiving a Pap test include having less than a high school education, being Hispanic, being low income, and being currently uninsured [12-14].

More than 1.1 million people in the United States are living with HIV infection and almost 1 in 6 (15.8%) are unaware of their infection [15]. Although women only represent approximately 25% of people living with a diagnosis of HIV infection, women of color are disproportionately represented [16]. At some point in their lifetimes, an estimated 1 in 32 black/African American women will be diagnosed with HIV infection, compared with 1 in 106 Hispanic/Latino women and 1 in 526 White women [16].

At some point in their lives, most sexually active people will contract an STI [17,18], the most common of which are human papilloma virus (HPV), chlamydia, and gonorrhea. It has been estimated that about half to nearly all sexually-active men and women will get at least one type of HPV at some point in their lives [19,20]. While not all HPV infections will result in cervical cancers, HPV is the main cause of cervical cancer [21].

In 2011, the overall rate of chlamydial infection among women in the United States was more than twice the rate for males [19], and this was an increase of 36.2% for males and 20.2% increase for females since 2007 [19]. Racial disparities for chlamydia and gonorrhea are striking: the rate of infection for chlamydia and gonorrhea among blacks was 7 and 17 times higher than the rates for whites [19]. Among all races and both sexes, the highest rates of chlamydia and gonorrhea infection were among teens and young adults between the ages of 15–24 years old [19]. While this age group only represented approximately 25% of the sexually experienced population, they account for half of all new STIs in the United States [22].

### Sexual and reproductive health service delivery

In the United States, men, women, and teens rely on a mix of private and public providers of sexual and reproductive health services, including ~15,000 private practice obstetrician-gynecologists [23], ~75,000 office-based family practice doctors [24], and ~8,400 publicly-funded clinics [25]. Roughly half (49%) of these publicly-funded clinics provided sexual and reproductive health services using funding from the federal Title X Family Planning Program.

#### Title X Family Planning Program

In the United States, the Title X Family Planning Program was created in 1970 to fund the provision of sexual and reproductive health services for men, women, and teens through a network of public and private nonprofit health and community service agencies throughout the country. The program also funds training for clinic personnel, research and evaluation, information dissemination, and community-based education and outreach [2]. While all clients are eligible to receive services, the Title X program prioritizes services to individuals from low-income families [26] through a sliding fee scale that includes 100% subsidization of services for clients who are at or below 100% of federal poverty guidelines [27]. Title X regulations state that family planning services must be provided without regard to age (See 42 CFR 59 § 59.5) and that such services are confidential (See 42 CFR 59 § 59.11), which means teens can receive family planning services in Title X-funded clinics without parental notification, although guidelines encourage family participation in decision-making [27].

In fiscal year 2012, the Title X program received approximately $296.8 million in federal funding. Combined with client fees, fees from 3^rd^ party payers (e.g., private health insurance and Medicaid), and revenue from other sources collected in 2012, the program funded services for almost 4.8 million people in 4,189 sites throughout all 50 United States, the District of Columbia, and eight US territories and jurisdictions [28]. During 2012, 8.9% of the clients were under 18 years old, 51.0% were 20–29 years old, 20.3% were 30–39 years old, and 9.2% were 40 years and older; 91.9% percent were female; and 20.4% were Black/African American, 55.9% were White, and 28% were Hispanic or Latino [28].

#### The Patient Protection and Affordable Care Act

Signed into law on March 23, 2010, the Patient Protection and Affordable Care Act (ACA) has increased access to sexual and reproductive health services, mostly for women who are covered by private health insurance, through a series of health insurance reforms. Starting six months after enactment, non-grandfathered health insurance plans must have provided preventative services rated A or B by the US Preventive Services Task Force with no cost sharing to consumers. The sexual and reproductive health services on this list currently include breast and cervical cancer screening, chlamydial infection screening (for sexually active women under 25 and all higher risk women), gonorrhea screening (for all higher risk women), HIV screening (everyone 15–65 years old, pregnant, and higher risk), STD counseling (for all sexually active adolescents and higher-risk adults), and syphilis screening (for pregnant women and adults at higher risk) [29]. Additionally, starting August 1, 2012, non-grandfathered health insurance plans must have provided certain women’s preventive services with no cost sharing to consumers. Those sexual and reproductive health services currently include HPV testing (for women 30 years and older), counseling for STIs (for all women), and counseling and screening for HIV (for all sexually active women).

For low-income men and women, the ACA provided states with incentives to cover these services in their Medicaid program and to expand their Medicaid program to include all low-income people up to 138% of the federal poverty level; however due to a recent ruling by the United States Supreme Court, the Medicaid expansion is now optional for states. Revised estimates project that Medicaid coverage will be expanded to include 13 [30] to 18 [31] million more Americans, for a new total of approximately 78 million Medicaid enrollees nationwide by 2023 [31]; all of whom will benefit from the expanded coverage for sexual and reproductive health services. However, because not all states will expand their Medicaid plans and due to gaps and exceptions in the law, it is expected that 31 million Americans will still lack health insurance after full implementation of the ACA [30].

### Preferences for providers of sexual and reproductive health services

Title X funding is aimed at increasing affordable access to sexual and reproductive health services for low-income people; thus, low-income men, women, and teens constitute the majority of clients served by Title X-funded clinics. For many low-income people, Title X-funded providers are the only source of ongoing health care and health education related to sexual and reproductive health [32]. Of the 6.7 million women estimated to have received contraceptive services in publicly-funded clinics, 70% of them received care at sites supported by the Title X program [25]. In spite of this large number of clients served, however, there is a surprising dearth of scientific studies examining the reasons why clients seek services at Title X-funded clinics over other health care providers.

In 1994, Sugerman and colleagues [33] surveyed 356 adolescents and 424 adults in three Planned Parenthood clinics in Los Angeles County and found that the primary reasons for using the family planning clinic over other health care providers was cost and confidentiality. Similarly, in 2011 and 2012, Frost and colleagues [34] surveyed 2,094 women at 22 family planning clinics in 13 states and found that accessibility, positive staff interactions, affordability, contraception method availability, and confidentiality were “very important” to their choice to visit a Title X-funded specialized family planning clinic over another health care provider.

However, given that more sexual and reproductive health services will be available to more low-income people in the coming years as a result of the Affordable Care Act, it is unclear if Title X-funded clinics will still be needed. To help inform policy debates regarding the on-going need for Title X-funded services, a research study was conducted to gather the perceptions of and preferences for Title X-funded family planning clinics using a convenient sample of clients receiving services in Northeast Ohio.

## Methods

### Participants and setting

The participants in this study were clients who received sexual and reproductive health services at one of eight clinics receiving federal Title X family planning funding as a part of the Northeast Ohio Family Planning Program. These clinics included those run by health departments, Planned Parenthood, Federally Qualified Health Centers (FQHCs), or other independent non-profit organizations. In September 2012, all clients who received services were asked to complete a Client Satisfaction Survey before leaving the facility. Clients were told that if they completed the brief survey, they would be entered in a drawing to receive a $50 Visa gift card. IRB approval was received from Kent State University (#13-511) to conduct secondary data analysis of the surveys.

### Measures and methods

The survey asked for demographic information (age, sex, race/ethnicity, and home ZIP code), perceptions of the clinic, and preferences for where they obtain sexual and reproductive health services. The survey was printed in English and was self-administered using a pencil/pen and clipboard that was provided by the clinic.

#### Clinic perceptions

On a scale of 1 to 5, where 1 = poor and 5 = great, participants were asked how easy it was to get care at the clinic, the friendliness and resourcefulness of front desk staff, interactions with medical staff, cleanliness and comfort of the facility, affordability of services, and confidentiality of personal information.

#### Utilization preferences

On a scale of 1 to 5, where 1 = never go here to 5 = always go here, participants were asked where they go for birth control, STD testing, physical exams, pregnancy testing, health information, and HIV testing. In a separate question, participants were asked if they consider the clinic their primary source of health care.

#### Data entry and analysis

The data were entered using EpiInfo7 for PC and then exported into SPSS Version 22 for Mac for analysis. Descriptive statistics (frequencies, means, and standard deviations) were performed to describe the study sample and report participants’ clinic perceptions and utilization preferences. To determine statistically significant differences by gender, age, and race on utilization preferences, the Kruskal-Wallis test was performed due to concerns of normality in the data. Post-hoc comparisons were performed using Mann–Whitney for comparisons with more than two groups (e.g., race). Statistical significance was set to p < .05.

## Results

### Sample characteristics

A total of 696 clients completed the survey in eight clinics. During the time period of data collection (September 2012), there were a total of 2,645 clients who received sexual and reproductive health services and were offered the opportunity to complete the survey. The response rate to the survey ranged between 12.6% and 39.5% across the eight clinics, for an overall weighted response rate of 26.4%. Of the 696 surveys completed, 9% came from health departments, 19% from Planned Parenthood clinics, 8% from Federally Qualified Health Centers (FQHCs), and 64% from other independent non-profit organizations.

As shown in Table 1, the overall age range of respondents was 14–61 years of age. Most (54.6%) participants were between the ages of 20–29 years old, 19.5% were between 14–19 years old, 16.7% were between 30–39 years old, and 7.8% were 40 years or older. Ninety percent were adults and 8.3% were teens (up to 17 years old). Eighty eight percent of participants were female. Overall, 94.8% of female participants were women of reproductive age (female between 15–44 years old). Most (60.8%) participants were White (Not Hispanic or Latino), 21.7% were Black/African American (Non Hispanic or Latino), 7.9% were Hispanic (all Races), and 2.8% were another race.

**Table 1:** Descriptive statistics of study sample (n = 696)

**Characteristic**	**Frequency**	**Percent**
*Age*		
10-19 years old	136	19.5
20-29 years old	380	54.6
30-39 years old	116	16.7
40-49 years old	43	6.2
50-59 years old	9	1.3
60+ years old	2	0.3
Missing	10	1.4
*Teen/Adult*		
Teen (13–17 years old)	58	8.3
Adult (18+ years old)	628	90.2
Missing	10	1.4
*Sex*		
Male	79	11.4
Female	610	87.6
Missing	7	1.0
Woman of Reproductive Age (female 15–44 years old)	578	94.8^1^
*Race/Ethnicity*		
Asian	7	1.0
Pacific Islander	1	0.1
Black/African American (Not Hispanic or Latino)	151	21.7
American Indian/Alaska Native	6	0.9
White (Not Hispanic or Latino)	423	60.8
Hispanic (All Races)	55	7.9
Unknown/Other	5	0.7
Missing	48	6.9
Clinic is Regular Source of Health Care	512	73.6%

^1^out of 610 women.

### Perceptions of Title X-funded family planning clinics

Participants were asked to rate the clinic on a range of characteristics including ease of getting care, front desk and medical staff, the facility, and the affordability and confidentiality of services. As summarized in Table 2, the majority (between 62.1-71.1%) rated the ease of getting care at the clinic as “Great”. This included the ability to get an appointment when needed (71.1%), the hours the clinic is open (62.1%), and the convenience of the clinic’s location (69.1%). Most (83.3-83.5%) rated the front desk staff as “Great” on how friendly and helpful they were (83.5%) and how well they answer the client’s questions (83.3%). Most (between 84.6-87.2%) rated the medical staff as “Great” on the amount of time spent with the client (84.6%), the advice and treatment received (87.2%), how well they listen to the client (86.6%), and how well they explain what the client wants to know (85.5%). Most (between 80.2-84.3%) rated the facility as “Great” for cleanliness (84.3%), comfort (80.9%), and privacy in waiting and exam rooms (80.2%). Most rated the cost of services (81.0%) and confidentiality (90.4%) as “Great”. Lastly, nearly three quarters (73.6%) considered the Title X-funded family planning clinic as their regular source of health care.

**Table 2:** Frequencies, means, and standard deviations of self-reported perceptions of clinics (n = 696)

**Perceptions**	**Poor**	**Fair**	**Okay**	**Good**	**Great**	**Missing**
	*f* (%)	*f* (%)	*f* (%)	*f* (%)	*f* (%)	*f* (%)
*Ease of Getting Care*						
Ability to get an appointment when you need one	0 (0.0)	4 (0.6)	48 (6.9)	145 (20.8)	**495 (71.1)**	4 (0.6)
Hours clinic is open	5 (0.7)	11 (1.6)	47 (6.8)	192 (27.6)	**432 (62.1)**	9 (1.3)
Convenience of clinic’s location	4 (0.6)	8 (1.1)	44 (6.3)	143 (20.5)	**481 (69.1)**	16 (2.3)
						
*Front Desk Staff*						
Friendly and helpful to you	0 (0.0)	1 (0.1)	14 (2.0)	97 (13.9)	**581 (83.5)**	3 (0.4)
Answers your questions	0 (0.0)	0 (0.0)	12 (1.7)	96 (13.8)	**580 (83.3)**	8 (1.1)
						
*Medical Staff*						
Takes enough time with you	1 (0.1)	0 (0.0)	12 (1.7)	91 (13.1)	**589 (84.6)**	3 (0.4)
Gives you good advice and treatment	1 (0.1)	2 (0.3)	8 (1.1)	75 (10.8)	**607 (87.2)**	3 (0.4)
Listens to you	2 (0.3)	0 (0.0)	10 (1.4)	76 (10.9)	**603 (86.6)**	5 (0.7)
Explains what you want to know	2 (0.3)	1 (0.1)	6 (0.9)	86 (12.4)	**595 (85.5)**	6 (0.9)
						
*Facility*						
Neat and clean	0 (0.0)	1 (0.1)	8 (1.1)	98 (14.1)	**587 (84.3)**	2 (0.3)
Comfortable	0 (0.0)	2 (0.3)	21 (3.0)	106 (15.2)	**563 (80.9)**	4 (0.6)
Privacy in waiting and exam rooms	1 (0.1)	4 (0.6)	26 (3.7)	99 (14.2)	**558 (80.2)**	8 (1.1)
						
*Affordability*						
Cost of services	0 (0.0)	4 (0.6)	30 (4.3)	88 (12.6)	**564 (81.0)**	10 (1.4)
						
*Confidentiality*						
Keeping my personal information private	0 (0.0)	0 (0.0)	11 (1.6)	52 (7.5)	**629 (90.4)**	4 (0.6)

Bold indicates majority of response.

### Preferences for utilizing Title X-funded family planning clinics

Participants were asked to indicate where they usually go for common sexual and reproductive health services, including birth control, STD testing, physical exams, pregnancy testing, health information, and HIV testing. As shown in Table 3, most stated they “always” go to the Title-X funded family planning clinic for birth control (61.4%), STD testing (61.6%), pregnancy testing (50.3%), and HIV testing (56.6%). Less than half, but still the most respondents, “always” go to the Title X-funded family planning clinics for physical exams (49.9%) and health information (47.6%).

**Table 3:** Frequencies, means, and standard deviations of self-reported preferences for utilizing participating clinics for sexual and reproductive health services (n = 696)

**Sexual and Reproductive Health Service**	**Never**	**Sometimes**	**No Preference**	**Usually**	**Always**	**Missing**
	*f* (%)	*f* (%)	*f* (%)	*f* (%)	*f* (%)	*f* (%)
Birth Control	49 (7.0)	11 (1.6)	38 (5.5)	42 (6.0)	**427 (61.4)**	129 (18.5)
STD Testing	37 (5.3)	13 (1.9)	43 (6.2)	51 (7.3)	**429 (61.6)**	123 (17.7)
Physical Exams	76 (10.9)	25 (3.6)	56 (8.0)	62 (8.9)	**347 (49.9)**	130 (18.7)
Pregnancy Testing	65 (9.3)	21 (3.0)	57 (8.2)	45 (6.5)	**350 (50.3)**	158 (22.7)
Health Information	53 (7.6)	28 (4.0)	70 (10.1)	72 (10.3)	**331 (47.6)**	142 (20.4)
HIV Testing	51 (7.3)	14 (2.0)	47 (6.8)	51 (7.3)	**394 (56.6)**	139 (20.0)

Bold indicates majority of response.

As summarized on Table 4, females preferred to utilize the Title X-funded family planning clinic more than males for birth control, physical exams, pregnancy testing, and health information, with no gender differences for STD and HIV testing. By age, teens preferred to utilize the Title X-funded family planning clinic more than adults for STD and HIV testing, physical exams, pregnancy testing, and health information, with no age difference for birth control services. There were no statistically significant racial or ethnic differences except for physical exams, where Whites preferred to use the Title X-funded family planning clinic for this service more than Blacks.

**Table 4:** Mean ranks of preferences for using Title X-Funded family planning clinics for sexual and reproductive health services by gender, age, and race/ethnicity using the Kruskal-Wallis test

	**Gender**			**Age**			**Race**				**Post-Hoc**
	***Male***	***Female***	***p-value***	***Teen***	***Adult***	***p-value***	***Black***	***Hispanic***	***White***	***p-value***	**Comparisons**^**1**^
Birth Control	151.78	**290.64**	0.000*	309.95	277.86	0.087	236.42	271.29	260.14	0.106	
STD Testing	286.87	284.22	0.876	**324.73**	280.02	0.022*	250.76	269.36	259.91	0.618	
Physical Exams	215.48	**286.85**	0.001*	**323.33**	276.12	0.030*	227.96	271.19	263.71	0.025*	W > B
Pregnancy Testing	151.28	**273.90**	0.000*	**307.58**	262.34	0.029*	233.50	268.68	241.75	0.312	
Health Information	230.84	**279.13**	0.024*	**320.58**	269.93	0.021*	239.83	251.79	253.96	0.584	
HIV Testing	282.23	275.87	0.726	**314.07**	272.15	0.036*	236.40	256.80	255.35	0.297	

*p < .05.

^1^Post-hoc comparisons using Mann–Whitney.

Bold indicates statistical significance at p<.05.

## Discussion

### Perceptions of Title X-funded family planning clinics

Emerging research on effective patient-provider communication emphasizes a number of important characteristics, including providers that provide good advice and treatment [35-37], take enough time with their patients [38], listen to their patients [39-42], and explain what their patients need to know [37,39,42-49]. These effective patient-provider communication techniques have been linked to a number of outcomes, including cancer screening among women [50,51], efficacy of treatment plans [52], adolescents’ decisions to seek health care [53], and adolescents’ intentions to keep follow-up appointments [54]. In this study, clients rated the perceptions of the Title X family clinics very positively, with some of the highest ratings for interactions with medical staff. These findings are consistent with Frost and colleagues [34], who also found that accessibility, positive staff interactions, affordability, contraception method availability, and confidentiality were “very important” to their choice to visit a Title X-funded specialized family planning clinic over another health care provider. The high rankings in these areas indicate that providers in these Title X-funded family planning clinics are exhibiting strong and effective patient-provider communication, which can help to enable positive outcomes, especially for the women and adolescents that prefer to use these facilities for their sexual and reproductive health needs.

### Preferences for utilizing Title X-funded family planning clinics

One of the most important sexual and reproductive health needs for female adults and teens is affordable access to contraception. Rates of unintended pregnancy are highest and have increased for women age 20–24 [3], especially those with lower levels of education and income [3]. In this study, female clients reported a significant preference for Title X-funded family planning clinics over other clinics for birth control services and pregnancy testing. Teens also reported a significant preference for Title X-funded family planning clinics for pregnancy testing, likely because of the access and privacy protections afforded to teens in Title X clinics. Although the survey did not directly measure income level, 85% of the clients served by these Title X-funded family planning clinics had incomes at or below 150% of the federal poverty level in 2012. The high self-reported preferences for Title X-funded clinics for birth control services and pregnancy testing among these predominantly low income clients is a strong signal that these Title X-funded clinics may be the best setting to deliver affordable access to contraception for low-income women and teens.

Regular physical exams that include clinical breast exams and Pap tests are important to detecting breast and cervical cancer early, which can decrease mortality. While overall breast cancer incidence is highest among White Non-Hispanic women, it is higher among African American women under 40 years of age [6]. Incidence of cervical cancer is also higher among African American women, as well as Hispanics. Death rates for breast cancer and cervical cancer is also higher among minority women and is largely due to delayed detection, having less education, being low-income, and being uninsured [6,10-14]. These results showed that the majority of clients surveyed preferred to receive physical exams in Title X-funded family planning clinics over other settings, with a significant preference among women and teens. Among racial/ethnic groups, only Non-Hispanic Whites reported stronger preferences than Non-Hispanic Blacks for physical exams at Title X-funded clinics and this was the only statistically significant racial/ethnic-based difference in all sexual and reproductive health services. The survey did not measure frequency of receiving physical exams, including clinical breast exams or Pap tests, so actual utilization cannot be assessed, only preference for location. One interpretation of these findings is that while there is strong preference for Title X-funded clinics overall and among women and teens for physical exams, the lower rankings among African Americans, Hispanics, and adults suggest that these Title X-funded family planning clinics need to improve perceptions among these populations, since these groups experience disproportionate incidence and mortality outcomes for breast cancer and cervical cancer.

The rate of HIV infection among 20–24 year olds is higher than any other age group and has increased in recent years [55]. Additionally, although they represent only 25% of the population, teens and young adults aged 15–24 account for nearly half of all new STD infections [22]. This study showed that most clients preferred to access STD and HIV testing services from Title X-funded family planning clinics over other clinics. The only two sexual and reproductive health services that did not have statistically significant gender-based differences were STD and HIV testing. Given the much higher mean rank scores for males for both of these services compared to other services, the lack of significance is likely due to ceiling effects. There were also no statistically significant race/ethnicity-based differences in preferences for places to receive STD and HIV testing services. However, there were much stronger preferences among teens for these services. Given the high rates of STDs and HIV in this younger population, this research suggests that these Title X-funded family planning clinics are preferred locations for these screening services among this high-risk group, particularly because these services can be provided in Title X clinics confidentially without parental notification.

### Need for Title X-funded family planning clinics

The majority of clients who participated in the research reported very favorable perceptions of Title X-funded family planning clinics and strong preferences for receiving sexual and reproductive health services at these facilities. Preferences for Title X-funded family planning clinics was strongest among females and teens for the majority of sexual and reproductive health services, indicating that these clinics are successfully reaching these high risk populations. Given that the majority of these clients are low-income, these findings suggest that these Title X-funded family planning clinics are uniquely positioned to provide sexual and reproductive health services to populations at highest risk for unintended pregnancy and STD/HIV infection.

Although the ACA will increase access to sexual and reproductive health services for many people, most of the changes will benefit women who already have health insurance or low income women who will become eligible if they live in a state that expands its Medicaid program to a level which allows them to qualify. Given that an estimated 31 million people will still be uninsured after the full implementation of the ACA [30] and because lack of health insurance is a major barrier to accessing health care and a determinant of negative sexual and reproductive health outcomes, these findings suggest that the Title X Family Planning Program and the clinics it funds should remain in place for the foreseeable future.

### Limitations

This study reported perceptions of and utilization preferences for Title X-funded family planning clinics using a sample of clients that received sexual and reproductive health services from eight clinics in Northeast Ohio that received Title X funding. The overall response rate for the survey was 26.4%, which is comparable to other similar voluntary surveys, but exposes the study to self-selection bias. In addition, the response rate was not equal among the different types of family planning providers and most of the surveys came from clients who received services at independent non-profit organizations. Clients responded to the survey inside the clinic so participants could have been biased toward providing socially desirable responses; however, the anonymity of the survey mitigates its potential effect. Also, there was a higher level of non-response to questions regarding the utilization preferences of sexual and reproductive health services, which was likely due to respondents not turning over the survey to finish it.

The study also attempts to help inform policy debates regarding the on-going need for Title X-funded services given increased access to sexual and reproductive health services resulting from the Affordable Care Act. While these findings demonstrate strong preferences for the Title X-funded clinics over other sources of health care, it is important to note that the survey did not directly measure the need and value of the Title X program compared to other locations where sexual and reproductive health services could be obtained. It also did not assess clinical outcomes. Lastly, and most importantly, this survey was only administered to clients in Title X-funded family planning clinics in Northeast Ohio, so the results may not be indicative of perceptions and preferences of Title X clients outside of Northeast Ohio or any other user of sexual and reproductive health services. Readers should consider these limitations when interpreting these findings.

## Conclusions

The majority of clients who participated in the research reported very favorable perceptions of Title X-funded family planning clinics and strong preferences for receiving sexual and reproductive health services at these facilities. It also demonstrated that these clinics successfully reached populations in need of sexual and reproductive health services and consequently, can help play a significant role in reducing disparities in these groups even after full implementation of the Affordable Care Act. However, due to its limitations, more research is needed to fully quantify the need and value of Title X-funded family planning clinics and its relation to the changing health care environment in the US.

## Competing interests

The author declares that he was the former director of the Northeast Ohio Family Planning Program.

## Authors’ contributions

The author developed the survey, analyzed the results, drafted the manuscript, and is accountable for all aspects of the research.
